# Empathy and Person-Centered Care from the perspective of undergraduate Speech-Language Pathology students

**DOI:** 10.1590/2317-1782/e20240380en

**Published:** 2025-09-09

**Authors:** Giovana Fochezato Veloso, Tatiane Franciele de Almeida, Vanessa Luisa Destro Fidêncio

**Affiliations:** 1 Clínica-Escola de Fonoaudiologia, Universidade Tuiuti do Paraná – UTP - Curitiba (PR), Brasil.; 2 Programa de Pós-graduação em Saúde da Comunicação Humana, Universidade Tuiuti do Paraná – UTP - Curitiba (PR), Brasil.

**Keywords:** Speech, Language and Hearing Sciences, Students, Patient-centered Care, Empathy, Surveys and Questionnaires

## Abstract

**Purpose:**

To correlate the empathy of undergraduate Speech-Language Pathology students with their preference for the person-centered care (PCC) model.

**Methods:**

This is a cross-sectional quantitative study using a questionnaire. Undergraduate Speech Language Pathology students from any academic year, enrolled in any Higher Education Institution (HEI), aged 18 years or older, participated in the study. Participants completed the Brazilian Portuguese version of the Patient-Practitioner Orientation Scale (PPOS) and the Empathy Inventory via an online form. A simple descriptive statistical analysis and data analysis based on total scores and dimensions of the applied instruments were performed. A significance level of p < 0.05 was adopted.

**Results:**

Forty undergraduate Speech-Language Pathology students participated, with an average age of 26.25 years. Of these, 82.5% were students from private HEIs, and 67.5% were from the southern region of the country. Regarding the PPOS scale, participants scored higher in the “caring” dimension. For the Empathy Inventory, higher scores were observed in the “affective sensitivity” factor and lower scores in the “interpersonal flexibility” factor. A significant positive correlation was found between the total scores of the questionnaires, between the “interpersonal flexibility” factor and the “caring” dimension, and between the “altruism” factor and the “caring” dimension.

**Conclusion:**

In the evaluated sample, undergraduate Speech-Language Pathology students with higher levels of empathy, particularly altruism and interpersonal flexibility, demonstrated a tendency to prefer the PCC model. Thus, investing in strategies to enhance empathy in undergraduate students may encourage the adoption of the PCC model, contributing to improved patient care quality in Speech-Language Pathology.

## INTRODUCTION

Patient-centered care was promoted as an effort to recognize the individual in opposition to a paternalistic biomedical model. Although both are used as synonyms, evidence pointed out that patient-centered care and person-centered care have different goals, with the former aiming at a functional life, while the latter aims to provide a significant life for the assisted individual^(1)^. Considering that one does not oppose the other, in the current study, person-centered care (PCC) will be used.

PCC is organized around people’s needs and health expectations, not around diseases, promoting care based on mutual respect and collaboration between patient and professional^(2)^. Thus, the focus is on the person rather than on his/her medical conditions^(3)^. The PCC directly influences the quality of care^(3)^, and it is influenced by the existing disparities in health care^(4)^.

Considering the biopsychosocial care model, health professional education must encompass not only technical competencies, but also behavioral competencies^(5)^. Therefore, regarding Speech Therapy graduation, the undergraduate is expected to have humanistic education, becoming a sensitive professional focused on collective responsibility, among other competencies and skills^(6)^. However, even with the need for humanized care in the area, there is scarcity of studies that disclose whether speech therapists really apply PCC principles, the impact of the implementation of this type of care on clinical practice^(7)^, and the professional satisfaction with this model of caring^(8)^.

According to Lee and Ihm^(9)^, patient-centered attitude may and must be taught. Thus, future professionals’ education is deemed necessary regarding relational aspects in patient-speech therapist’s collaboration^(7)^.

In a study^(10)^ conducted with Nursing undergraduates, the researchers found that changes in self-awareness and active listening must be the first aspects to be trained among that population in order to favor PCC. However, there is scarce evidence on the factors associated with the preference for the PCC among Speech Therapy undergraduates.

Being questioned about the expectations of PCC delivery, patients pointed out the importance of communication and empathy^(11,12)^. Empathy stands out as one of the aspects encompassing PCC, along with respect, engagement, relationship, communication, shared decision-making, holistic focus, individualized focus and coordinated care^(1)^. It is the skill to put oneself in the other’s shoes, feeling what the other feels^(1,13)^, and that is one of the factors that affects patient-centered care^(14)^.

Despite patients’ differing preferences on their health professionals’ relationship in several aspects according to their culture, regarding the PCC aspects, empathy is acknowledged as a shared value among individuals from different countries^(15)^. Evidence suggests that improving the level of empathy is beneficial to enhance patient’s health care standards and quality of life^(16)^. Thus, it is important to study this aspect. A quick literature search found a single study^(17)^, which aimed to assess empathy among Speech Therapy undergraduates in Brazil. The authors reported that the mentioned population evidenced high levels of dispositional or trait empathy, tending to a decrease in such levels during the last two years of graduation

Confronting the urgent need to encourage health care systems to adopt an integrated and person-centered care approach in order to organize their services^(2)^, in the face of the need to elaborate strategies still in the health care graduation level, and given the lack of evidence on the theme among the population of Speech Therapy undergraduates, the current study aimed to correlate empathy of Speech Therapy undergraduates with their preference to the person-centered care model (PCC).

## METHODS

### Ethical aspects and study design

It is a cross-sectional, quantitative study with questionnaire application, held at Tuiuti University of Parana (Universidade Tuiuti do Paraná). The study began after its approval by the Ethics Research Committee of the aforementioned university, opinion number 6.524.733. The participants attested their participation by means of the Free Informed Consent Form.

### Sample selection

The following criteria were adopted for participant inclusion: 18 years of age and above, to be a Speech Therapy undergraduate attending any school terms at any HEIs in Brazil, and attest his/her participation by means of the Free Informed Consent Form. Participants were excluded for not answering all the mandatory questions of the data collection instruments.

Participants’ selection was conducted by using the “Virtual Snowball” sampling. In this technique, data collection begins with the release of a link to access the study questionnaire on the virtual social networks^(18)^. In the current study, Whatsapp® and Instagram® were used. In order to promote the study, apart from the research presentation, a request for sharing the link with the contact network was also inserted.

### Data collection instrument

The data collection instrument was provided in the form of an electronic formulary, elaborated in the Google Forms. On the first page, the Free Informed Consent Form was displayed. The individual only could read and respond to the questionnaires after accepting the Free Informed Consent Form. On the second page of the form, the questions regarding the participant’s profiling data and HEI (Higher Education Institution) were viewed. On the third and fourth pages, the data collection instruments were presented.

Initially, information was collected on birth date, age, gender, city and State of the HEI where the participant attended the Speech Therapy course, school term, and whether the participant had already attended a class about the PCC theme. Subsequently, the participants answered a version of the Patient-Practitioner Orientation Scale (PPOS) in Brazilian Portuguese, also known as *Escala de Orientação Médico-Paciente* ^(19)^.

The PPOS assesses patients, doctors and health students on the doctor-patient relationship, whether centered on the professional (biomedical model) or centered on the patient (psychosocial model). It comprises 18 items reflecting two dimensions: sharing and caring. The items regarding sharing (1, 4, 5, 8, 9, 10, 12, 15 and 18) assess to what extent professionals should share power (information and decisions) with their patients. The items concerning the caring dimension reflect if the respondents consider patients’ expectations, feelings and emotions as critical elements^(19)^.

PPOS scores were rated by means of a Likert scale, which ranged from 1 (totally agree) to 5 (totally disagree). For the analysis, low scores mean a practitioner-centered orientation (focus on biomedical issues), while high scores indicate preference to patient-centered caring (shared control, focus on the person). The mean of the scores to all items (total scoring), as well as to the nine items of each domain was calculated. Statements of items 9, 13 and 17 have reversed scores.

Besides the PPOS^(19)^, the participants also responded to the Empathy Inventory^(20)^. The Empathy Inventory was designed and validated to assess empathy. It comprises 40 items, divided in four factors, according to Chart 1.

**Chart 1:** Amount of items and factors assessed by the Empathy Inventory^(20)^

**Factor**	**Items**	**What does it assess?**
**Perspective taking**	12	Capacity to understand the perspective and feelings of the other, especially in situations of conflict of interest
**Interpersonal flexibility**	10	Capacity to tolerate behaviors, attitudes and thoughts of others. Low scoring in the interpersonal flexibility means difficulty in accepting different points of view and tendency to get easily upset by conflicting situations.
**Altruism**	9	Capacity of individuals to sacrifice their own interests in order to help others. Low scoring in this factor unveils an egotistic tendency.
**Affective sensitivity**	9	Feeling of compassion and interest in the emotional state of the other.

Source: the author

From the 40 items of the Empathy Inventory, 17 are reversed (3, 4, 5, 8, 9, 13, 16, 19, 20, 22, 24, 26, 30, 32, 35, 38 e 40), indicating that the responses to these items must be reversed to reach the final scoring^(20)^.

### Data analysis

Simple descriptive statistical analysis and data analysis for the total scoring and PPOS dimensions (sharing and caring), apart from the factors assessed in the Empathy Inventory were assessed. Shapiro-Wilk test was also carried out in order to test data normality. Subsequently, Spearman Correlation Test was applied for the correlations comprising PPOS total scoring, PPOS caring dimension and the interpersonal flexibility factor of the Empathy Inventory. For the correlation between the other domains of both questionnaires, Pearson Correlation Test was used. Significance level of p<0,05 was adopted.

## RESULTS

Forty (40) Speech Therapy undergraduates participated in the study, being 36 females and 4 males, mean age of 26.25 years. From these, 82.5% attended private HEIs. Concerning the region, 67,5% (n=27) were HEI undergraduates from the Southern region, 17.5% (n=7) from the Southeastern region, and 15% (n=6) from the Midwestern region. Among the participants, 21 reported attending classes on PCC, and 19 reported that they had never attended specific classes on the theme. In Table 1, descriptive statistics on the participants’ gender, age and attending school terms (semesters) are shown for groups.

**Table 1:** Descriptive Statistics regarding gender, age and academic term attended by the participants

		**N**	**%**
**Gender**	**Female**	36	90
	**Male**	4	10
**Age**	**18-20 years**	2	5
	**21-25 years**	24	60
	**26-30 years**	5	12.5
	**31-35 years**	4	10
	**36-40 years**	3	7.5
	**Over 41 years**	2	5
**Attended academic term (semester)**	**1st-2nd**	1	2.5
	**3rd-4th**	4	10
	**5th-6th**	16	40
	**7th-8th**	19	47.5

**Caption:** n = number of participants

In the PPOS, participants showed higher scores in the caring dimension. As for the Empathy Inventory, higher scores were observed in the affective sensitivity factor, while lower scores were observed in the interpersonal flexibility factor (Table 2).

**Table 2:** Simple descriptive Statistics of the scoring of the questionnaires PPOS and EI

		**Mean**	**Standard deviation**	**Minimum**	**Maximum**
**Age (years)**		26.25	7.469	19	53
**PPOS**	**Sharing**	3.64	0.709	2	4.77
	**Caring**	4.02	0.597	2.22	4.88
	**Total**	3.83	0.530	2.38	4.61
**EI**	**PT**	3.70	0.727	1.75	4.92
	**IF**	3.19	0.566	1.80	4.10
	**AL**	3.47	0.588	2.22	4.56
	**AS**	4.16	0.599	2	5
	**Total**	3.62	0.411	2.62	4.55

**Caption:** PPOS = Patient-Practitioner Orientation Scale; EI = Empathy Inventory; PT = Perspective Taking; IF = Interpersonal Flexibility; AL = Altruism; AS = Affective Sensitivity

In the correlation between the results of the PPOS and the Empathy Inventory, positive correlation stands out between the total scoring of the questionnaires, between the interpersonal flexibility factor (Empathy Inventory) and caring (PPOS), and between the altruism factor (Empathy Inventory) and caring (PPOS) (Table 3).

**Table 3:** Correlation between the domains of the PPOS and EI

**Domains**	**Sharing (PPOS)**	**Caring (PPOS)**	**Total (PPOS)**	**PT (EI)**	**IF (EI)**	**AL (EI)**	**AS (EI)**	**Total (EI)**	
**Sharing (PPOS)**	-	-	-	-	-	-	-	-
**Caring (PPOS)**	0.045*	-	-	-	-	-	-	-
**Total (PPOS)**	<0.001*	0.019*	-	-	-	-	-	-
**PT (EI)**	0.463	0.681	0.459	-	-	-	-	-
**IF (EI)**	0.509	0.030*	0.064	-	-	-	-	-
**AL (EI)**	0.477	0.006*	0.013*	0.470	-	-	-	-
**AS (EI)**	0.455	0.341	0.266	<0.001**	0.543	0.916	-	-
**Total (EI)**	0.206	0.021*	0.019*	<0.001**	0.003*	<0.001**	<0.001**	-

* Inferring analysis by means of Spearman’s correlation. Statistical difference at p<0.05;

** Inferring Analysis by means of Pearson’s Correlation. Statistical difference at p<0.05

**Caption:** PPOS = Patient-Practitioner Orientation Scale; EI = Empathy Assessment Index; PT = Perspective taking; IF = Interpersonal Flexibility; AL = Altruism; AS = Affective Sensitivity

## DISCUSSION

The current study aimed to correlate the empathic traits of Speech Therapy undergraduates whether they prefer or not the PCC. It was observed that 47.5% of the participants reported that they had never attended a class on PCC. Person-centered caring is assumedly addressed in Speech Therapy graduation courses in different disciplines, as the National Curricular Guidelines claim comprehensive, humanized education, with the development of relational competencies and socioemotional skills regarding patient’s preferences, values and expectations. However, it is questioned if that explicitly occurs under that denomination and in specific classes. Thus, the assumption is raised that participants had already attended classes on PCC, even if they reported something otherwise.

In the PPOS, the participants in the current study had higher scores in the caring dimension. This finding contradicts a study^(21)^ which assessed 93 Speech Therapy undergraduates at a university in Texas/USA who had higher scores in the sharing dimension. On the other hand, a study published in 2019^(22)^, with undergraduates from four courses of the health area, including 50 Speech Therapy students, also identified higher scores in the caring dimension, that is, a similar result observed in research conducted in Korea with Dentistry undergraduates. It is important to consider that cultural differences impact on preferences to the PCC^(15)^. Still, in the current study, the higher scoring found in the caring dimension may point to a difficulty on the part of the Speech Therapy participants in sharing information with patients, evidencing professional-centered caring, even though they consider individuals’ emotions and expectations.

In the Empathy Inventory, the highest scores were observed in the affective sensitivity factor, which reflects the feeling of compassion, while the lowest scores were found in the interpersonal flexibility factor, associated with the capacity for tolerating behaviors and attitudes of others. Mendes et al.^(23)^ applied the Empathy Inventory to 193 Nursing undergraduates and obtained similar results. Until the completion of the current study, only one study^(17)^ was found, which aimed to assess empathy among Speech Therapy undergraduates in Brazil. In it, by applying the Interpersonal Reactivity Index (IRI), the author reported lower scores in the personal anxiety domain, which has to do with the tendency to feel affliction or discomfort in response to the affliction of the other.

Reversed correlation between empathy traits and the attended academic term was observed in Nursing undergraduates^(23)^ as well as in Speech Therapy undergraduates^(17)^. Moura^(17)^ divided her sample of Speech Therapy undergraduates in two groups, as follows: pre-clinical (students attending the first and second years), and clinical (students from the third and fourth years). Results showed significantly higher scores in the pre-clinical group in the IRI subscales of perspective taking and empathic consideration. The author pointed out that there was no way to infer the practical implications of this result due to the lack of standardization for the assessed population. However, in the same study, differences between the groups were not observed by applying the Jefferson Scale of Physician Empathy – student version (JSPE-S). The author stressed that the JSE-HPS was not still validated for Brazilian students at the time of the study. Thus, further studies are recommended to consider the variations in the results according to the instrument used for data collection.

In the current study, no correlation was possible to establish between the academic term and the empathic traits due to the limitation of the sample, which mostly entailed undergraduates from the last two years of the Speech Therapy graduation course.

### Relation between empathy and Person-Centered Care

In the current study, a positive, meaningful correlation was observed between the total scoring of the PPOS and the total scoring of the Empathy Inventory, suggesting that Speech Therapy undergraduates with more empathy tend to prefer patient-centered health care. Empathy is the cornerstone in the relationship between health professionals and patients^(24)^, being one of the main contributors to establish trust in such a relationship^(21)^ and, therefore, strongly associated with the competency for PCC^(9,25)^.

Dockens, Bellon-Harn and Manchaiah^(21)^ reported that the caring dimension in the PPOS could be associated with empathy among Speech Therapy undergraduates in Texas, a finding also observed in the current study. In the analysis for domains of the used questionnaires, positive, meaningful correlation was observed between interpersonal flexibility and altruism factors in the Empathy Inventory and the caring dimension of the PPOS. This result suggests that Speech Therapy undergraduates with empathic traits, marked by greater interpersonal flexibility and altruism, tend to consider patients’ expectations, feelings and emotions as critical elements, which may favor the choice for PCC.

According to Zimmer-Gembeck^(26)^, individuals that report more flexibility present higher access to coping strategies, better skill to organize stress, more cognitive control over emotion and greater capacity for acting. Moreover, they are more capable of decentralization (to detach from the inner experience), use more engagement responses and fewer coping responses to the other. As for altruism, it features an emotional state, which essentially fosters one’s well-being. Altruistic people act towards others’ benefit rather than in their own interests^(27)^. Thus, it is not surprising that such traits are correlated with PCC among Speech Therapy undergraduates, as they may directly affect professional-patient relationship.

### Perspectives for Speech Therapy teaching

In order for future Speech Therapy professionals to adopt the PCC model among their patients, it is fundamental, as the National Curricular Guidelines claim, to develop behavioral competencies, such as empathy, apart from technical skills. Petrucci et al.^(24)^ highlight that empathy is a trait of a stable personality, and can be modified along the time by means of educational interventions. In that sense, understanding its evolution along the academic trajectory may foster strategy implementation by HEIs in order to enhance and keep the levels of empathy among their undergraduates^(21,24)^.

Educational programs must prioritize empathy development, emphasizing positive attitudes regarding communicative skills learning, and guiding educational follow-up sessions, preventing students from becoming less patient-centered as they advance in the course^(9)^. Moreover, those strategies must fundamentally be based on active methodologies, adjusted to meet the traits of a new student generation^(25,28)^.

In a literature review^(28)^, 16 studies proposing strategies for the development of empathy, published between 2018 and 2019, were included. Among the interventions, group discussions, training of communication skills, workshops with ethical dilemmas and realistic simulations stood out. None of the included studies was carried out with Speech Therapy undergraduates.

Van Melis et al.^(29)^ proposed a Social Skills Training (SST) to 22 Speech Therapy undergraduates enrolled in the 2nd year of a HEI from the interior of São Paulo State/Brazil. The training integrated the syllabus of the Psychology discipline, which prepares students for the supervised clinical training, and included 15 meetings, two hours each. The authors identified difficulties in communicative and empathic skills, which were enhanced with the SST, suggesting that this approach can be included in Speech Therapy courses from other HEIs. Jeon and Choi^(14)^ point out that strategies promoting empathy and self-efficacy in communication are necessary to strengthen PCC competency. Apart from that, by educating patients on the possibilities of their engagement, and elucidating their role in the health care process, they begin to understand better their proper levels of participation. Still, it is equally necessary to provide health professionals with specific training in PCC and interpersonal skills to facilitate patient engagement in caring^(30)^.

Therefore, to the extent that students experience the professional-patient relationship in practice, they can develop deeper understanding of patients’ role in the clinical process. Active participation, in this context, does not usually happen until postgraduation level^(21)^. Thus, further studies, longitudinal design^(22)^, are deemed necessary to follow up such evolution.

In the current context of discussion on the update of the National Curricular Guidelines for the Speech Therapy course, and considering the new generation of undergraduates entering the HEIs, the perspective of the Speech Therapy teaching is believed to lead to the refinement of cross-cutting competencies. This focus is essential to fit future professionals’ education into the contemporary demands of the health area. Resolution number 568/2017 from the National Health Council highlights the importance of educating professionals who are committed to the tenets of integrality, equity and humanization, which must be present in teaching since graduation^(5)^. Thus, it is fundamental that Speech Therapy curricula integrate cross-cutting competencies, such as empathy, effective communication and teamwork, which are essential to deliver humanized care and collaborative work within multiprofessional teams. Such competencies are essential for training professionals that understand their patients as a whole.

### Limitations of the study

PCC entails several facets, including aspects that cannot be directly compared. Therefore, studying the preference for the PCC among the populations is challenging^(15)^.

The current study uncovers some limitations. One of them is its cross-sectional design, which prevents the analysis of changes in the characteristics of empathy along the Speech Therapy graduation course. Given the possibilities of changes in the levels of empathy in the different academic terms, it is important to conduct longitudinal studies. Another limitation is the reduced sampling size and the absence of representativeness from different HEIs, academic terms and regions over the country. Thus, the obtained results must not be generalized. Therefore, further studies are encouraged, with larger samplings and more representative of Speech Therapy undergraduates from Brazil. Moreover, it should be pointed out that self-reported empathy cannot directly reflect empathic behavior in clinical practice^(16)^. Further studies are suggested to consider that factor and propose a more comprehensive assessment of this competency.

### Implications for the practice

As human beings, in their several dimensions, are the central focus of health area professions, it is indispensable to expand research on empathy beyond concentrated studies on Nursing and Medicine undergraduates, prevalent in the literature on the theme^(28)^. Despite the limitations, the current study addresses an updated and relevant theme, and it is among the few ones that investigate the empathy traits among Brazilian Speech Therapy undergraduates. In addition, until its completion, it was the first to propose the correlation between those traits and the preference to PCC in this population.

Those initial results are expected to encourage further studies on the theme and motivate HEIs to implement strategies aiming at the development of empathy skills among Speech Therapy undergraduates. Such efforts contribute to educating more humanized professionals, according to the National Curricular Guidelines, thus fostering quality health care.

## CONCLUSION

In the assessed sampling, Speech Therapy undergraduates, with empathy traits marked by altruism and interpersonal flexibility, showed a tendency to prefer the PCC model. Thus, investing in the empathy development during graduation may foster the adoption of the PCC, contributing to enhance the quality of care to Speech Therapy patients.
