# Supplementary material for: Empathy and Person-Centered Care from the perspective of undergraduate Speech-Language Pathology students
Source: Codas. 2025 Sep 9;37(5):e20240380. doi: 10.1590/2317-1782/e20240380en (PMC12490509; doi:10.1590/2317-1782/e20240380en)
Supplement: Translation

# Translation: Empatia e Cuidado Centrado na Pessoa na perspectiva de estudantes de graduação em Fonoaudiologia

**DOI:** 10.1590/2317-1782/e20240380pt

**Authors:** Giovana Fochezato Veloso, Tatiane Franciele de Almeida, Vanessa Luisa Destro Fidêncio

**Affiliations:** 1 Clínica-Escola de Fonoaudiologia, Universidade Tuiuti do Paraná – UTP - Curitiba (PR), Brasil.; 2 Programa de Pós-graduação em Saúde da Comunicação Humana, Universidade Tuiuti do Paraná – UTP - Curitiba (PR), Brasil.

**Keywords:** Fonoaudiologia, Estudantes, Assistência Centrada no Paciente, Empatia, Inquéritos e Questionários

## Abstract

**Objetivo:**

Correlacionar a empatia de estudantes de graduação em Fonoaudiologia com a preferência pelo modelo de cuidado centrado na pessoa (CCP).

**Método:**

Trata-se de um estudo transversal quantitativo com aplicação de questionário. Participaram estudantes de graduação em Fonoaudiologia, de qualquer período, matriculados em qualquer Instituição de Ensino Superior (IES), com idade a partir de 18 anos. Os participantes responderam à versão no português brasileiro da escala Patient-Practitioner Orientation Scale (PPOS) e ao Inventário de Empatia, por meio de formulário on-line. Foi realizada análise estatística descritiva simples e a análise dos dados por escore total e dimensões dos instrumentos aplicados. Adotou-se nível de significância de p<0,05.

**Resultados:**

Participaram 40 estudantes de graduação em Fonoaudiologia, com média de idade de 26,25 anos, sendo 82,5% estudantes de IES privadas e 67,5% da região Sul do país. Com relação à escala PPOS, os participantes apresentaram maiores escores na dimensão cuidar. Já no Inventário de Empatia, observaram-se maiores escores no fator sensibilidade afetiva e menores escores no fator flexibilidade interpessoal. Houve correlação positiva significativa entre o escore total dos questionários, entre o fator flexibilidade interpessoal e o cuidar e entre o fator altruísmo e o cuidar.

**Conclusão:**

Na amostra avaliada, os estudantes de graduação em Fonoaudiologia com características de empatia de maior altruísmo e flexibilidade interpessoal apresentaram uma tendência a preferir o modelo de CCP. Assim, investir em estratégias para o aprimoramento da empatia nos estudantes de graduação pode favorecer a opção pelo CCP, contribuindo para melhorar a qualidade no cuidado ao paciente na Fonoaudiologia.

## INTRODUÇÃO

O cuidado centrado no paciente lançou-se como um esforço para reconhecer o indivíduo em oposição a um modelo biomédico paternalista e, embora utilizados como sinônimos, evidências pontuaram que o cuidado centrado no paciente e o cuidado centrado na pessoa têm objetivos diferentes, sendo que o primeiro visa proporcionar uma vida funcional, enquanto o segundo visa proporcionar uma vida significativa para o indivíduo assistido^(1)^. Considerando que um não se opõe ao outro, no presente estudo será utilizado o termo cuidado centrado na pessoa (CCP).

O CCP está organizado em torno das necessidades e expectativas de saúde das pessoas e não das doenças, promovendo o cuidado baseado no respeito mútuo e na colaboração entre o paciente e o profissional^(2)^. Coloca-se assim, menos foco nas condições médicas e mais foco na pessoa^(3)^. O CCP exerce influência direta na qualidade do cuidado^(3)^ e é influenciado pelas disparidades existentes no cuidado em saúde^(4)^.

Considerando o modelo de cuidado biopsicossocial, a formação do profissional de saúde deve englobar, além das competências técnicas, as competências comportamentais^(5)^. Assim, no que diz respeito à graduação em Fonoaudiologia, espera-se que o discente tenha uma formação humanista e torne-se um profissional que apresente, dentre outras competências e habilidades, sensibilidade para voltar-se à responsabilidade coletiva^(6)^. No entanto, mesmo com a necessidade de humanização do cuidado na área, faltam estudos que explorem se os fonoaudiólogos realmente aplicam os princípios do CCP, quais os efeitos da implementação deste tipo de cuidado na prática clínica^(7)^ e a satisfação do profissional com esse modelo de cuidado^(8)^.

De acordo com Lee e Ihm^(9)^, a atitude centrada no paciente pode e deve ser ensinada. Faz-se necessária, portanto, a educação de futuros profissionais no que diz respeito a aspectos relacionais da colaboração paciente-fonoaudiólogo^(7)^.

Em um estudo^(10)^ realizado com estudantes de graduação em enfermagem, os pesquisadores constataram que, para essa população, mudanças na autoconsciência e na escuta ativa devem ser os primeiros aspectos a serem trabalhados para favorecer o CCP. Ressalta-se, no entanto, que faltam evidências a respeito dos fatores associados à preferência pelo CCP em alunos de graduação em Fonoaudiologia.

Quando questionados a respeito das expectativas quanto a um atendimento pautado no CCP, pacientes ressaltaram a importância da comunicação e empatia^(11,12)^. A empatia é evidenciada como um dos aspectos que compõem o CCP, assim como o respeito, engajamento, relacionamento, comunicação, tomada de decisão compartilhada, foco holístico, foco individualizado e cuidados coordenados^(1)^. Trata-se da habilidade de colocar-se no lugar do outro, sentir o que o outro sente^(1,13)^, e é um dos fatores que afetam a centralidade no paciente^(14)^.

Apesar das preferências dos pacientes no relacionamento com o profissional da saúde diferirem em vários aspectos de acordo com a cultura em que estão inseridos, dentre os aspectos do CCP, a empatia compõe valores comuns a indivíduos de diferentes países^(15)^. Evidências sugerem que melhorar o nível de empatia é benéfico para melhorar os padrões de cuidados de saúde e a qualidade de vida do paciente^(16)^. Assim, é importante que esse aspecto seja mais estudado. Em busca rápida na literatura foi encontrado apenas um estudo^(17)^ cujo objetivo foi avaliar a empatia de estudantes de graduação em Fonoaudiologia no Brasil. Os autores referiram que essa população relatou altos níveis de empatia disposicional, com tendência de diminuição desses níveis nos dois últimos anos de graduação.

Diante da necessidade urgente de encorajar os sistemas de saúde a adotarem uma abordagem de cuidados integrada e centrada na pessoa para organização dos serviços^(2)^, da necessidade da elaboração de estratégias ainda no ensino da graduação em saúde e da falta de evidências a respeito da temática na população de estudantes de graduação em Fonoaudiologia, o objetivo do presente estudo foi correlacionar a empatia de estudantes de graduação em Fonoaudiologia com a preferência pelo modelo de cuidado centrado na pessoa (CCP).

## MÉTODO

### Aspectos éticos e desenho do estudo

Trata-se de um estudo transversal quantitativo, com aplicação de questionário, realizado na Universidade Tuiuti do Paraná. O estudo teve início após aprovação do Comitê de Ética em Pesquisas da referida Universidade, sob parecer nº 6.524.733. Os participantes atestaram sua participação por meio do aceite do Termo de Consentimento Livre e Esclarecido (TCLE).

### Seleção da amostra

Para a inclusão dos participantes foram adotados os seguintes critérios: ter idade a partir de 18 anos; ser estudante de curso de graduação em Fonoaudiologia, de qualquer período, em qualquer IES do Brasil; atestar sua participação no estudo por meio do aceite do TCLE. Foram excluídos do estudo: participantes que não responderam a todas as questões obrigatórias dos instrumentos de coleta.

A seleção dos participantes ocorreu pelo tipo de amostragem “Bola de Neve Virtual”. Nesse método, inicia-se a coleta de dados pela divulgação do link para acesso ao questionário do estudo por meio das redes sociais virtuais (RSV)^(18)^, sendo utilizadas, no presente estudo, *Whatsapp®* e *Instagram®*. Na divulgação, além da apresentação da pesquisa, foi inserida uma solicitação para que o link fosse compartilhado com a rede de contatos.

### Instrumento de coleta

O instrumento de coleta foi disponibilizado em formato de formulário eletrônico, elaborado no *Google Forms*. Na primeira página, foi apresentado o TCLE. O indivíduo apenas poderia realizar a leitura e preenchimento dos questionários após o aceite do TCLE. Na segunda página do formulário, foram apresentadas as questões referentes aos dados gerais do participante e IES. Na terceira e quarta página foram apresentados os instrumentos de coleta.

Inicialmente, foram coletadas informações sobre data de nascimento, idade, sexo, cidade e estado da IES em que o participante cursava Fonoaudiologia, período cursado e se o participante já havia assistido a uma aula sobre a temática de CCP. Em seguida, os participantes responderam a versão no português brasileiro da escala *Patient-Practitioner Orientation Scale* (PPOS), também conhecida como Escala de Orientação Médico-Paciente^(19)^. No presente estudo, será utilizada a sigla PPOS.

A escala PPOS avalia a percepção de pacientes, médicos e estudantes da área da saúde a respeito da relação médico-paciente, quando centrada no profissional (modelo biomédico) ou quando centrada no paciente (modelo psicossocial). Consiste em 18 itens que refletem duas dimensões: “compartilhar” (*sharing*) e “cuidar” (*caring*). Os itens referentes à dimensão do compartilhar (1, 4, 5, 8, 9, 10, 12, 15 e 18) avaliam o quanto os profissionais deveriam partilhar o poder (informações e decisões) com seus pacientes. Os itens referentes à dimensão do cuidar refletem se os respondentes consideram como elementos críticos as expectativas, os sentimentos e as emoções dos pacientes^(19)^.

Os escores da PPOS foram obtidos por meio de uma escala *Likert* que variou de 1 (concordo totalmente) a 5 (discordo totalmente). Para análise, considerou-se que baixos escores refletem uma orientação centrada no profissional (enfoque nas questões biomédicas) e altos escores indicam preferências por um cuidado mais centrado no paciente (controle compartilhado, enfoque na pessoa). Calculou-se a média das pontuações de todos os itens (escore total) e para os nove itens de cada domínio. Destaca-se que as afirmativas dos itens 9, 13 e 17 apresentam escores invertidos.

Além da PPOS^(19)^, os participantes também responderam ao Inventário de Empatia^(20)^. O Inventário de Empatia trata-se de um instrumento construído e validado para avaliar a empatia. É composto por 40 itens, divididos em quatro fatores, conforme disposto no Quadro 1.

**Quadro 1:** Quantidade de itens e fatores avaliados no Inventário de Empatia^(20)^

**Fator**	**Itens**	**O que avalia?**
**Tomada de perspectiva**	12	Capacidade em entender a perspectiva e os sentimentos da outra pessoa, especialmente em situações de conflito de interesse
**Flexibilidade interpessoal**	10	Capacidade para tolerar comportamentos, atitudes e pensamentos dos outros. Uma baixa pontuação na flexibilidade interpessoal indica dificuldade em aceitar pontos de vista diferentes e tendência em se aborrecer facilmente em situações conflituosas.
**Altruísmo**	9	Capacidade do indivíduo em sacrificar os próprios interesses com a finalidade de beneficiar ou ajudar o outro. Um escore baixo nesse fator revela uma tendência egoísta
**Sensibilidade afetiva**	9	Sentimento de compaixão e interesse pelo estado emocional do outro.

Fonte: elaboração própria

Dos 40 itens do Inventário de Empatia, 17 são inversos (3, 4, 5, 8, 9, 13, 16, 19, 20, 22, 24, 26, 30, 32, 35, 38 e 40), indicando que as respostas a esses itens devem ser invertidas para a obtenção do escore final^(20)^.

### Análise dos dados

Foi realizada análise estatística descritiva simples e a análise dos dados por escore total e dimensões do PPOS (compartilhar e cuidar), e dos fatores avaliados no Inventário de Empatia. Realizou-se o teste de *Shapiro-Wilk* para verificar a normalidade dos dados. Após, para as correlações que envolviam o escore total do PPOS, a dimensão cuidar do PPOS e o fator flexibilidade interpessoal do Inventário de Empatia, aplicou-se o teste de Correlação de *Spearman*. Para a correlação entre os demais domínios de ambos os questionários, utilizou-se o teste de Correlação de *Pearson*. Adotou-se nível de significância de p<0,05.

## RESULTADOS

Participaram do estudo 40 estudantes de graduação em Fonoaudiologia, sendo 36 do sexo feminino e 4 do sexo masculino, com média de idade de 26,25 anos. Destes, 82,5% eram estudantes de IES privadas. Com relação à região, 67,5% (n=27) eram estudantes de IES na região Sul do país, 17,5% (n=7) da região Sudeste e 15% (n=6) da região Centro-Oeste. Dos participantes, 21 afirmaram já terem assistido a aulas sobre o CCP e 19 afirmaram nunca terem assistido a aulas específicas sobre o assunto.Na tabela 1 está disposta a estatística descritiva referente ao sexo, idade e período cursado pelos participantes, por grupos.

**Tabela 1:** Estatística descritiva referente ao sexo, idade e período cursado pelos participantes

		**n**	**%**
**Sexo**	**Feminino**	36	90
	**Masculino**	4	10
**Idade**	**18-20 anos**	2	5
	**21-25 anos**	24	60
	**26-30 anos**	5	12,5
	**31-35 anos**	4	10
	**36-40 anos**	3	7,5
	**Acima de 41 anos**	2	5
**Período cursado (semestre)**	**1º-2º**	1	2,5
	**3º-4º**	4	10
	**5º-6º**	16	40
	**7º-8º**	19	47,5

**Legenda:** n = nº de participantes

Na escala PPOS, os participantes apresentaram maiores escores na dimensão cuidar. Já no Inventário de Empatia, observaram-se maiores escores no fator sensibilidade afetiva e menores escores no fator flexibilidade interpessoal (Tabela 2).

**Tabela 2:** Estatística descritiva simples dos escores dos questionários PPOS e IE

		**Média**	**Desvio-padrão**	**Mínimo**	**Máximo**
**Idade (anos)**		26,25	7,469	19	53
**PPOS**	**Compartilhar**	3,64	0,709	2	4,77
	**Cuidar**	4,02	0,597	2,22	4,88
	**Total**	3,83	0,530	2,38	4,61
**IE**	**TP**	3,70	0,727	1,75	4,92
	**FI**	3,19	0,566	1,80	4,10
	**AL**	3,47	0,588	2,22	4,56
	**SA**	4,16	0,599	2	5
	**Total**	3,62	0,411	2,62	4,55

**Legenda:** PPOS = *Patient-Practitioner Orientation Scale*; IE = Inventário de Empatia; TP = Tomada de Perspectiva; FI = Flexibilidade Interpessoal; AL = Altruísmo; SA = Sensibilidade Afetiva

Na correlação entre os resultados da escala PPOS e do Inventário de Empatia, destaca-se correlação positiva entre o escore total dos questionários, entre o fator flexibilidade interpessoal (Inventário de Empatia) e o cuidar (escala PPOS) e entre o fator altruísmo (Inventário de Empatia) e o cuidar (escala PPOS) (Tabela 3).

**Tabela 3:** Correlação entre os domínios do PPOS e do IE

**Domínios**	**Compartilhar (PPOS)**	**Cuidar (PPOS)**	**Total (PPOS)**	**TP (IE)**	**FI (IE)**	**AL (IE)**	**SA (IE)**	**Total (IE)**	
**Compartilhar (PPOS)**	-	-	-	-	-	-	-	-
**Cuidar (PPOS)**	0,045*	-	-	-	-	-	-	-
**Total (PPOS)**	<0,001*	0,019*	-	-	-	-	-	-
**TP (IE)**	0,463	0,681	0,459	-	-	-	-	-
**FI (IE)**	0,509	0,030*	0,064	-	-	-	-	-
**AL (IE)**	0,477	0,006*	0,013*	0,470	-	-	-	-
**SA (IE)**	0,455	0,341	0,266	<0,001**	0,543	0,916	-	-
**Total (IE)**	0,206	0,021*	0,019*	<0,001**	0,003*	<0,001**	<0,001**	-

* Análise inferencial pela Correlação de Spearman, diferença estatística para p<0,05;

** Análise inferencial pela Correlação de Pearson, diferença estatística para p<0,05

**Legenda:** PPOS = *Patient-Practitioner Orientation Scale*; IE = Inventário de Empatia; TP = Tomada de Perspectiva; FI = Flexibilidade Interpessoal; AL = Altruísmo; SA = Sensibilidade Afetiva

## DISCUSSÃO

O objetivo do presente estudo foi correlacionar as características de empatia de estudantes de graduação em Fonoaudiologia com a preferência ou não pelo CCP. Observou-se que 47,5% dos participantes afirmaram nunca terem assistido a uma aula sobre o CCP. Acredita-se que o CCP seja abordado nos cursos de graduação em Fonoaudiologia nas diferentes disciplinas, visto que as Diretrizes Curriculares Nacionais (DCN)^(6)^ enfatizam uma formação abrangente e humanizada, com o desenvolvimento de competências relacionais e habilidades socioemocionais e o respeito às preferências, valores e expectativas do paciente. Entretanto, questiona-se se isso ocorre explicitamente com essa denominação e em aulas específicas. Dessa forma, levanta-se a hipótese de que, apesar de terem afirmado o contrário, os participantes possivelmente já tenham assistido a aulas sobre o CCP.

Na escala PPOS, os participantes do presente estudo obtiveram escores mais elevados na dimensão cuidar. Esse achado diverge do encontrado em um estudo^(21)^ que avaliou 93 estudantes de graduação em Fonoaudiologia de uma Universidade do Texas, nos EUA, e obteve escores mais altos na dimensão compartilhar. Por outro lado, um estudo publicado em 2019^(22)^, envolvendo estudantes de quatro cursos da área da saúde, incluindo 50 estudantes de Fonoaudiologia, também identificou escores mais elevados na dimensão cuidar, resultado semelhante ao observado em uma pesquisa^(9)^ conduzida na Coréia, com estudantes de Odontologia. É importante considerar que diferenças culturais impactam nas preferências pelo CCP^(15)^. Ainda assim, acredita-se que o resultado de maior escore na dimensão cuidar no presente estudo indica uma dificuldade dos estudantes de graduação em Fonoaudiologia participantes em compartilhar informações com os pacientes, evidenciando um modelo de cuidado mais centrado no profissional, ainda que considere as emoções e expectativas do indivíduo.

No Inventário de Empatia, os maiores escores foram observados no fator sensibilidade afetiva, que reflete o sentimento de compaixão, enquanto os menores escores ocorreram no fator flexibilidade interpessoal, associado à capacidade para tolerar comportamentos e atitudes do outro. Mendes et al.^(23)^ aplicaram o Inventário de Empatia em 193 estudantes de graduação em enfermagem e obtiveram resultados semelhantes. Até a conclusão do presente estudo, apenas um estudo^(17)^ que se propôs a avaliar a empatia de estudantes de Fonoaudiologia no Brasil foi encontrado. Nele, ao aplicar a Escala Multidimensional de Reatividade Interpessoal (EMRI), a autora observou menores escores no domínio angústia pessoal, que diz respeito à tendência em sentir aflição ou desconforto em resposta à aflição do outro.

Tanto em estudantes de enfermagem^(23)^, quanto em estudantes de Fonoaudiologia^(17)^, foi observada correlação inversa entre características de empatia e o período acadêmico. Moura^(17)^ dividiu sua amostra de estudantes de graduação em Fonoaudiologia em dois grupos, sendo: pré-clínico (estudantes do 1º e 2º ano) e clínico (estudantes do 3º e 4º ano). Os resultados mostraram pontuações significativamente maiores no grupo pré-clínico nas subescalas tomada de perspectiva e consideração empática da EMRI. A autora destacou que não há como inferir sobre as implicações práticas desse resultado, devido à falta de normatização para a população avaliada. No mesmo estudo, entretanto, não foram observadas diferenças entre os grupos na aplicação da Escala Jefferson de Empatia Médica – versão para estudantes (JSPE-S). A autora ressaltou que, na época do estudo, a JSE-HPS ainda não havia sido validada para estudantes brasileiros. Assim, recomenda-se que estudos futuros considerem as variações nos resultados em função do instrumento utilizado para coleta de dados.

No presente estudo não foi possível estabelecer uma correlação entre o período acadêmico e as características de empatia devido à limitação na amostra, que foi composta, em sua maioria, por estudantes dos últimos dois anos do curso de graduação em Fonoaudiologia.

### Relação entre empatia e Cuidado Centrado na Pessoa

No presente estudo, observou-se uma correlação positiva e significativa entre o escore total da PPOS e o escore total do Inventário de Empatia, sugerindo que estudantes de Fonoaudiologia mais empáticos tendem a preferir a centralidade no paciente no cuidado em saúde. A empatia é a pedra angular do relacionamento entre profissionais de saúde e pacientes^(24)^, sendo um dos principais contribuintes para o estabelecimento de confiança nessa relação^(21)^, estando, portanto, fortemente associada à competência para o CCP^(9,25)^.

Dockens, Bellon-Harn e Manchaiah^(21)^ relataram que a dimensão cuidar da escala PPOS poderia estar associada à empatia em estudantes de Fonoaudiologia no Texas, um achado que também foi observado no presente estudo. Na análise por domínios dos questionários utilizados, observou-se uma correlação positiva significativa entre os fatores flexibilidade interpessoal e altruísmo do Inventário de Empatia e a dimensão cuidar da escala PPOS. Esse resultado sugere que os estudantes de Fonoaudiologia com características empáticas marcadas por maior flexibilidade interpessoal e altruísmo tendem a considerar como elementos críticos as expectativas, sentimentos e emoções dos pacientes, o que pode favorecer a escolha pelo CCP.

De acordo com Zimmer-Gembeck^(26)^, indivíduos que relatam mais flexibilidade apresentam maior acesso a estratégias de enfrentamento, melhor habilidade de organizar o estresse, mais controle cognitivo sobre a emoção e uma maior capacidade de agir. Além disso, são mais capazes de se descentralizar (de se desligar da experiência interna), usam mais respostas de engajamento e menos respostas de enfrentamento ao outro. Já o altruísmo caracteriza-se como um estado emocional, que tem como essência aumentar o bem-estar do outro. Pessoas altruístas agem em benefício do outro em vez de seus próprios interesses^(27)^. Assim, não surpreende o fato dessas características estarem correlacionadas ao CCP em estudantes de graduação em Fonoaudiologia, visto que podem impactar diretamente na relação profissional-paciente.

### Perspectivas para o ensino em Fonoaudiologia

Para que os futuros profissionais da Fonoaudiologia adotem o modelo de CCP com seus pacientes, é fundamental que, como preconizam as DCN^(6)^, sejam desenvolvidas, além das habilidades técnicas, as competências comportamentais, como a empatia. Petrucci et al.^(24)^ destacam que a empatia não é um traço de personalidade estável, podendo ser modificada ao longo do tempo por meio de intervenções educacionais. Nesse sentido, compreender sua evolução ao longo da trajetória acadêmica pode possibilitar que as IES implementem estratégias para aprimorar e sustentar os níveis de empatia entre os estudantes^(21,24)^.

Os programas educacionais devem priorizar o desenvolvimento da empatia, enfatizando atitudes positivas em relação ao aprendizado de habilidades comunicativas e conduzindo sessões educacionais de acompanhamento para evitar que os estudantes se tornem menos centrados no paciente à medida que avançam no curso^(9)^. Além disso, é essencial que essas estratégias sejam embasadas em metodologias ativas, ajustadas para atender às características de uma nova geração de estudantes^(25,28)^.

Em uma revisão de literatura^(28)^ foram incluídos 16 estudos publicados entre 2018 e 2019, que propuseram estratégias para o desenvolvimento da empatia. Entre as intervenções, destacaram-se as discussões em grupos, treinamento de habilidades de comunicação, oficinas com dilemas éticos e simulações realistas. Nenhum dos estudos incluídos havia sido realizado com estudantes de Fonoaudiologia.

Van Melis et al.^(29)^ propuseram um Treinamento de Habilidades Sociais (THS) para 22 estudantes de Fonoaudiologia matriculados no 2º ano do curso em uma IES no interior de São Paulo. O treinamento integrou o conteúdo programático da disciplina de Psicologia, voltada ao preparo dos alunos para o estágio clínico supervisionado, e incluiu 15 encontros de duas horas cada. As autoras identificaram dificuldades nas habilidades comunicativas e empáticas, que foram aprimoradas com o THS, sugerindo que essa abordagem pode ser incorporada em cursos de Fonoaudiologia de outras IES. Jeon e Choi^(14)^ destacam que estratégias que promovam a empatia e a autoeficácia da comunicação são necessárias para fortalecer a competência de CCP. Além disso, ressalta-se que, ao educar os pacientes sobre as possibilidades de envolvimento e esclarecer seu papel no processo do cuidado em saúde, eles passam a compreender melhor os níveis adequados de participação. Ainda assim, é igualmente necessário oferecer aos profissionais de saúde um treinamento explícito em CCP e habilidades interpessoais, a fim de facilitar o envolvimento dos pacientes no cuidado^(30)^.

Ressalta-se que, à medida que os estudantes vivenciam de forma prática a relação profissional-paciente, podem desenvolver uma compreensão mais profunda sobre o papel do paciente no processo clínico. Comumente, a participação ativa nesse contexto não ocorre até a formação em nível de pós-graduação^(21)^. Assim, faz-se necessária a realização de futuros estudos com delineamentos longitudinais^(22)^ para acompanhar essa evolução.

No contexto atual de discussão sobre a atualização das DCN para o curso de Fonoaudiologia e levando em consideração a nova geração de alunos que está ingressando nas IES, acredita-se que a perspectiva do ensino em Fonoaudiologia deva se direcionar também ao aprimoramento das competências transversais. Este enfoque é essencial para alinhar a formação dos futuros profissionais às demandas contemporâneas da área da saúde. A Resolução nº 568/2017, do Conselho Nacional de Saúde, destaca a importância da formação de profissionais comprometidos com os princípios da integralidade, da equidade e da humanização, os quais devem estar presentes no ensino desde a graduação^(5)^. Assim, é fundamental que os currículos de Fonoaudiologia integrem as competências transversais, como empatia, comunicação efetiva e trabalho em equipe, que são essenciais para o atendimento humanizado e para a atuação colaborativa em equipes multiprofissionais. Essas competências são indispensáveis para a formação de profissionais que compreendam o paciente de forma integral.

### Limitações do estudo

Diversas facetas compõem o CCP, incluindo aspectos que não podem ser diretamente comparados. Assim, estudar a preferência pelo CCP entre as populações é um desafio^(15)^.

O presente estudo apresenta algumas limitações. Uma delas é o seu delineamento transversal, que impossibilita a análise das mudanças nas características de empatia ao longo do curso de graduação em Fonoaudiologia. Dada a possibilidade de variações nos níveis de empatia em diferentes períodos acadêmicos, é importante que sejam conduzidos estudos longitudinais. Outra limitação é o tamanho reduzido da amostra e a ausência de representatividade de diferentes IES, períodos acadêmicos e regiões do país. Assim, os resultados obtidos não devem ser generalizados. Encoraja-se, portanto, que novos estudos sejam realizados com amostras maiores e mais representativas dos estudantes de Fonoaudiologia do Brasil. Além disso, ressalta-se que a empatia autorrelatada pode não refletir diretamente um comportamento empático no atendimento clínico^(16)^. Portanto, sugere-se que futuros estudos considerem também esse fator e proponham uma avaliação mais abrangente dessa competência.

### Implicações para a prática

Dado que o ser humano, em suas diversas dimensões, é o foco central das profissões da área da saúde, torna-se imprescindível ampliar as pesquisas sobre empatia para além dos estudos concentrados em alunos de Enfermagem e Medicina, que predominam na literatura sobre a temática^(28)^. Apesar das limitações, o presente estudo aborda uma temática atual e relevante, sendo um dos poucos a investigar as características de empatia de estudantes brasileiros de graduação em Fonoaudiologia. Ademais, até sua finalização, este foi o primeiro a propor a correlação entre essas características e a preferência pelo CCP nessa população.

Espera-se que esses resultados iniciais incentivem a realização de novos estudos sobre o tema e motivem as IES a implementar estratégias voltadas ao desenvolvimento das habilidades de empatia em estudantes de graduação em Fonoaudiologia. Esse esforço contribui para a formação de profissionais mais humanizados, em consonância com as DCN, favorecendo a qualidade do cuidado em saúde.

## CONCLUSÃO

Na amostra avaliada, os estudantes de graduação em Fonoaudiologia com características de empatia marcadas por maior altruísmo e flexibilidade interpessoal demonstraram uma tendência a preferir o modelo de CCP. Assim, investir no desenvolvimento da empatia ainda durante a graduação pode favorecer a adoção do CCP, contribuindo para a melhoria da qualidade no cuidado ao paciente na Fonoaudiologia.
